# *Luffa echinata* Roxb. Induces Human Colon Cancer Cell (HT-29) Death by Triggering the Mitochondrial Apoptosis Pathway

**DOI:** 10.3390/molecules17055780

**Published:** 2012-05-16

**Authors:** Li-Hua Shang, Chun-Mei Li, Zhao-Yang Yang, De-Hai Che, Jing-Yan Cao, Yan Yu

**Affiliations:** 1 Department of Internal Medicine, The Third Affiliated Hospital of Harbin Medical University, Haping Road 150 of Nangang District, Harbin 150081, China; 2 Department of Medical Biotechnology, Kangwon National University, Chuncheon, Gangwon 200-701, Korea

**Keywords:** *Luffa echinata* Roxb., apoptosis, Bax/Bcl-2, cell cycle, reactive oxygen species (ROS)

## Abstract

The antiproliferative properties and cell death mechanism induced by the extract of the fruits of *Luffa echinata* Roxb. (LER) were investigated. The methanolic extract of LER inhibited the proliferation of human colon cancer cells (HT-29) in both dose-dependent and time-dependent manners and caused a significant increase in the population of apoptotic cells. In addition, obvious shrinkage and destruction of the monolayer were observed in LER-treated cells, but not in untreated cells. Analysis of the cell cycle after treatment of HT-29 cells with various concentrations indicated that LER extracts inhibited the cellular proliferation of HT-29 cells via G2/M phase arrest of the cell cycle. The Reactive oxygen species (ROS) level determination revealed that LER extracts induced apoptotic cell death via ROS generation. In addition, LER treatment led to a rapid drop in mitochondrial membrane potential (MMP) as a decrease in fluorescence. The transcripts of several apoptosis-related genes were investigated by RT-PCR analysis. The caspase-3 transcripts of HT-29 cells significantly accumulated and the level of Bcl-XL mRNA was decreased after treatment with LER extract. Furthermore, the ratio of mitochondria-dependent apoptosis genes (Bax and Bcl-2) was sharply increased from 1.6 to 54.1. These experiments suggest that LER has anticancer properties via inducing the apoptosis in colon cancer cells, which provided the impetus for further studies on the therapeutic potential of LER against human colon carcinoma.

## 1. Introduction

Cancer is a serious clinical problem that poses enormous personal, clinical, and societal challenges to the healthcare system. Colorectal cancer (CRC) is the third most common cancer in both males and females and accounts for about 9% of cancer deaths each year. In 2008, 1.23 million new cases of colorectal cancer were clinically diagnosed, and it killed 608,000 people [1]. Several strategies have been applied to fight this cancer, including surgical resection, cytotoxic drugs, and radiation therapy [2]. A number of anti-cancer drugs have been isolated from plants [3,4,5]. There is an urgent need for new therapeutic agents for CRC patients. Apoptosis, as a physiological mode of cell death, is an intrinsic program and a key regulator of tissue homeostasis [6]. The imbalances between cell death and proliferation may result in tumor formation [7]. Thus, the objective of anti-cancer agent is to induce apoptosis-related signaling in cancer cells while disrupting their proliferation [8].

*Luffa echinata* Roxb. (LER), a slender herb which grows widely in the Bengal, Gujarat and Uttar Pradesh regions of India, is commonly known as “Bindal” in Hundi and belongs to the Cucurbitaceae [9]. Some of its biologically active compounds have already been reported, including amariin [10], echinatin, saponins [11], hentriacontane, gypsogenin [12], cucurbitacin B [13], datiscacin, 2-*O*-β-D-glucopyranosyl cucurbitacin-B and 2-*O*-β-D-glucopyranosyl cucurbitacin-S [14]. The therapeutic properties of LER have been comprehensively studied. LER has been reported to potentiate hypnosis in mice [15], and produce diuretic activity in dogs and cats [16]. LER showed some potential therapeutic effects on diabetes [17], jaundice, biliary and intestinal colic, and enlarged liver and spleen [18]. Furthermore, the fruits of LER have a curative effect on hypertension [19], chronic bronchitis, lung complaints and [9] and its hepatoproctive activity has been also reported [20].

The aims of this study were to evaluate the cytotoxic properties of an extract prepared from the fruits of LER and to determine the mechanism of cell death elicited by the extract in CRC cells. The human colon cancer cell (HT-29) is a cell line widely used as an experimental model for *in vitro* studies of many normal and neoplastic processes. Thus, we investigated the effects of the methanolic extract of LER fruits on HT-29 cells.

## 2. Results and Discussion

### 2.1. LER Inhibits the Proliferation of HT-29 Cells

**Figure 1:**
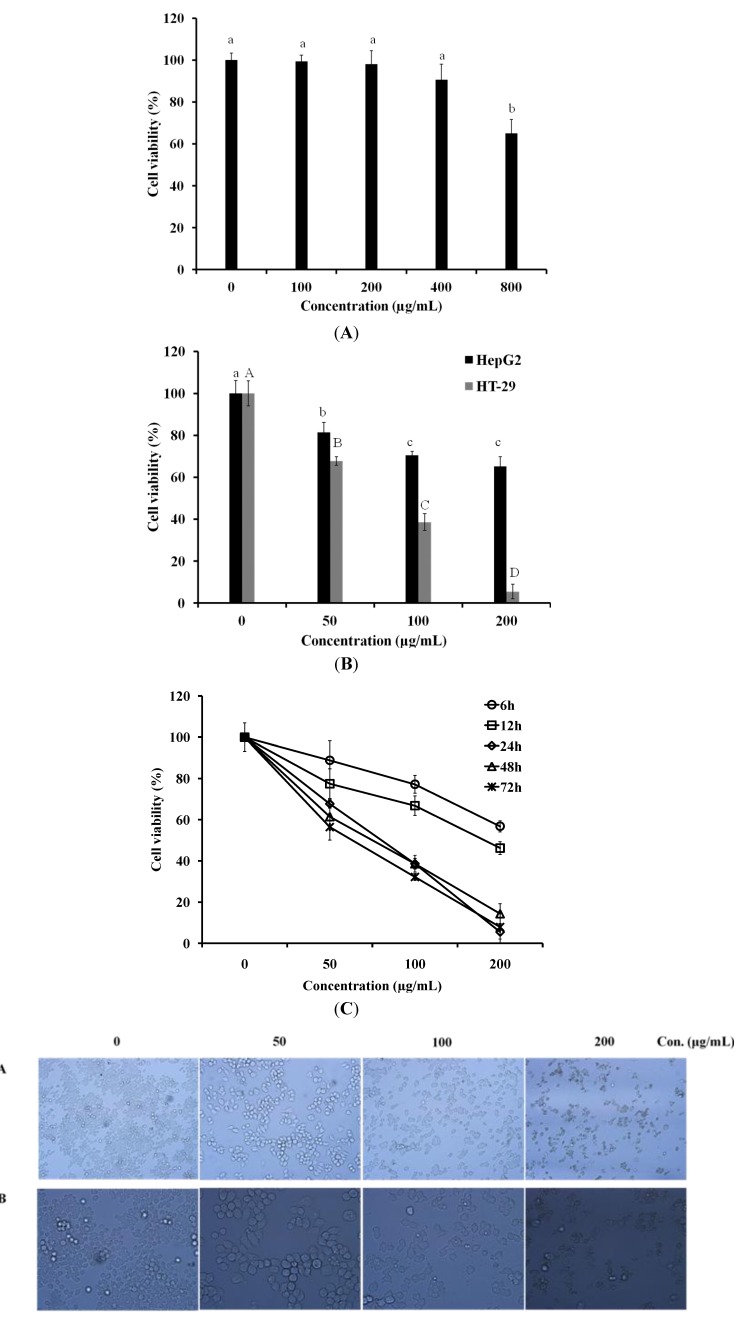
Cytotoxicity and anti-proliferative activity of *Luffa echinata*. (**A**) The CCD-986Sk cells were plated in 96-well plates and then exposed to 100, 200, 400, and 800 µg/mL of *L. echinata* extract for 24 h. With in a column, values with the same superscript letters are not significantly different from each other at *p* < 0.05; (**B**) HepG2 and HT-29 cells were plated in 96-well plates and then exposed to 50, 100, and 200 µg/mL of *L. echinata* extract for 24 h. The cell viability of HT-29 cells treated by *L. echinata* extract was measured using an MTT assay. ^a–c^ indicated the statistical significance of the anti-proliferative activity of various concentrations of LER extract on HepG2 cells (*p* < 0.05). ^A–D^ indicated the statistical significance on HT-29 (*p* < 0.05); (**C**) HT-29 cells were incubated with 50, 100, and 200 µg/mL of *L. echinata* extract for 6, 12, 24, 48, 72 h, respectively. The cell viability of HT-29 cells treated by *L. echinata* extract was measured by MTT assay. (**D**) Cell culture morphology was assessed by light microscopy after incubation with various concentrations of *L. echinata* extract for 24 h.

Before the investigation of anti-cancer activity of LER extract, the cytotoxicity of LER on normal cells was estimated using a cortical collecting duct (CCD-986Sk) cell line. LER showed low cytotoxicity on normal cells when its concentration was lower than 200 µg/mL (Figure 1A). In this study, human hepatocellular liver carcinoma cells (HepG2) cell line and human colon cancer cell line (HT-29) were used to examine the antiproliferative activity of LER extract by MTT assay (Figure 1B). Cultured HepG2 and HT-29 cells were treated with LER extracts for 24 h at the concentrations of 50, 100, and 200 µg/mL, respectively. LER extract showed no significant inhibition effect on HepG2 cells, however, LER dramatically inhibited HT-29 cell viability in a does-dependent manner. All concentrations of extracts exerted a significant inhibitory effect on the cells following 24 h of exposure to LER. Because the LER extract showed good anti-cancer activity on human colon cancer cell line, the anti-proliferative activity of LER extract on HT-29 cells was investigated further. Cultured HT-29 cells were treated with LER extract for 6, 12, 24, 48, and 72 h at the concentrations of 50, 100, and 200 µg/mL, respectively (Figure 1C). LER dramatically inhibited HT-29 cell viability in both dose-dependent and time-dependent manners. All concentrations of extracts exerted an inhibitory effect on the cells and the proliferation of HT-29 cells was reduce to 50% following 24 h of exposure to LER at 80.6 µg/mL. However, after 24 h, the changes in anti-proliferative effect of LER were not obviously different. Thus, the treatment with LER extract for 24 h was used as a model in further study.

To examine the effect of the methanol extract on cellular morphology during cell death, the morphological changes in untreated HT-29 cells and cells treated with LER extract at different concentrations for 24 h were analyzed by light microscopy (Figure 1D). A dose-dependent reduction in population size was noted in the LER-treated cells. Direct observation by microscopy revealed the cellular morphology was severely disordered in the cells treated by 200 µg/mL LER extract. Compared to the untreated cells, the LER-treated cells showed obvious cell shrinkage, destruction of the monolayer, and condensed chromatin which was not seen in the untreated control cells.

### 2.2. LER Extract Induces a G2/M Cell Cycle Arrest in HT-29 Cells

**Figure 2:**
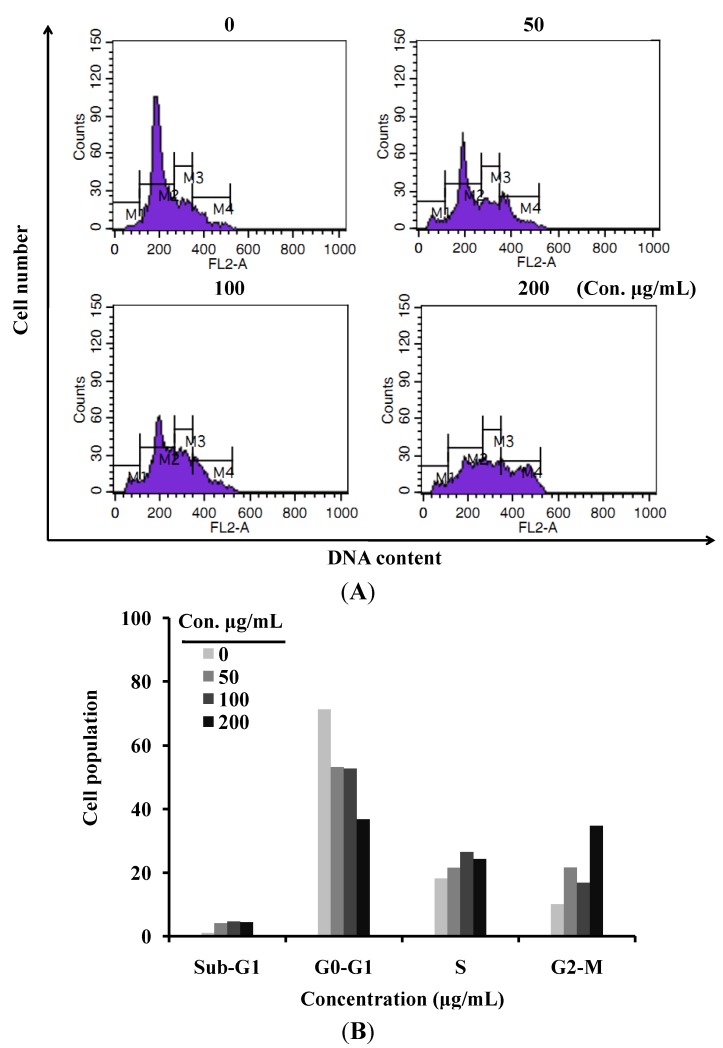
The effect of *L. echinata* on the HT-29 cell cycle. (**A**) Cells treated with different concentrations of *L.echinata* for 24 h and analyzed by flow cytometry after staining with PI; (**B**) Histogram showing the number of cells in each cell cycle.

Cell cytometry analysis was performed to investigate whether LER affected cell cycle regulation (Figure 2). From the result, 21.59, 16.77, and 34.71% of cells were arrested at G2/M phase after treatment with 50, 100, and 200 μg/mL of LER extract, respectively, whereas in the untreated group, 10.06% of HT-29 cells were in G2/M phase. The increase of cells at G2/M phase showed a dose-dependent trend with the treatment of LER extract and was mostly at the expense of the population of cells at G0/G1 phase. Analysis of the cell cycle after treatment of HT-29 cells with various concentrations of LER revealed that the percentage of the G0/G1 phase of the cycle decreased and the G2/M phase of cells increased, which indicated that LER extract inhibited the cellular proliferation of HT-29 cells via G2/M phase arrest of the cell cycle.

### 2.3. LER Extract Induces Cell Death by ROS Generation and Mitochondrial Membrane Potential (MMP) Lose

**Figure 3:**
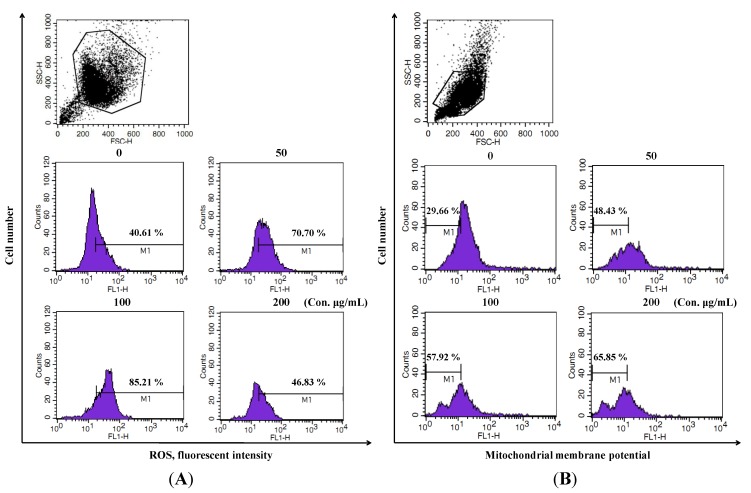
Effect of *L. echinata* on ROS and MMP levels in HT-29 cells. (**A**) Cells treated with different concentrations of *L. echinata* for 24 h and the ROS level analyzed by flow cytometry after staining with DCFH-DA; (**B**) The MMP level of the incubated cells were analyzed by flow cytometry after staining with DiOC_6_.

To determine whether the anti-proliferative effect of LER extract was mediated by the generation of reactive oxygen species (ROS), the ROS level in HT-29 cells was measured using DCFH-DA in a flow cytometry analysis system (Figure 3A). Compared to untreated control cells, the ROS levels in HT-29 cells were strongly increased after treatment with LER extract at various concentrations for 24 h. As shown in Figure 3A, the ROS generation in the HT-29 cells treated with 100 µg/mL of LER extract was sharply increased to 85.21%. Decreased fluorescence of 3,3'-dihexyloxacarbocyanine iodide (DiOC6) indicates a decrease in MMP and a loss of mitochondrial membrane integrity. FACS results from the treated/untreated HT-29 cells were shown in Figure 3B. Compared with the untreated group, levels of MMP in LER incubated groups were gradually decreased in the LER incubated cells in a dose-dependent manner (M1, 29.66% in untreated cells *versus* 48.43, 57.92, 65.85% in 50, 100, and 200 µg/mL respective treated HT-29 cells).

### 2.4. Expression of Apoptotic-Related Genes in LER Extract-Treated Cells

**Figure 4:**
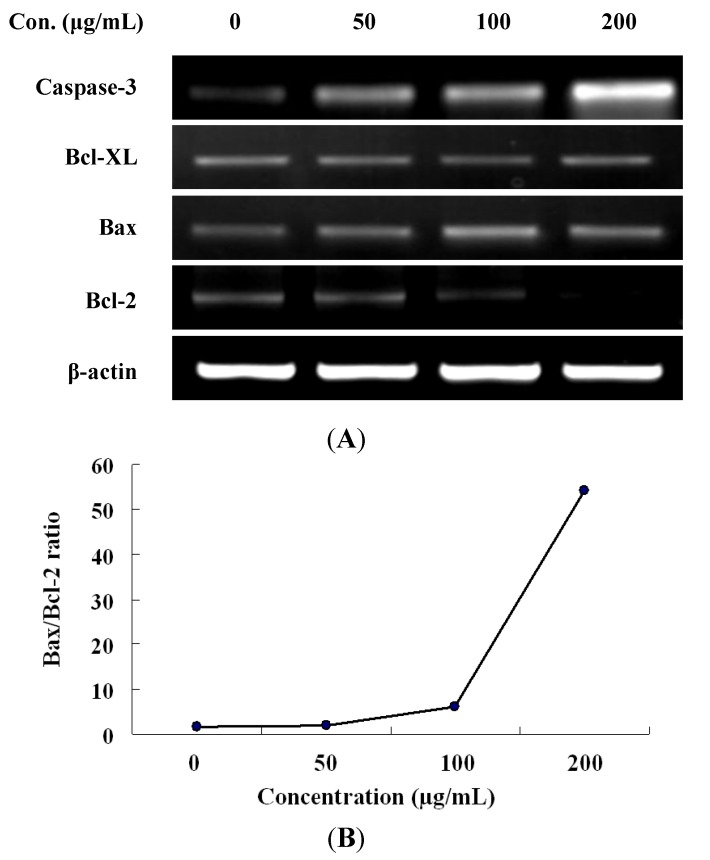
(**A**) RT-PCR anlysis for Bcl-2, Bax, Bcl-XL, and caspase-3 mRNA expression on *L. echinata* extract-stimulated HT-29 cells. Cells were plated in 6-well plates and incubated with *L. echinata* for 24 h; (**B**) The ratio of Bax to Bcl-2 was determined by densitometric analysis.

To investigate the possible molecular mechanism by which the LER extract triggered apoptosis in HT-29 cells, the expression of several apoptotic-related genes in HT-29 cells treated by LER was evaluated by RT-PCR with β-actin as an internal control (Figure 4). Compared to untreated cells, the caspase-3 transcripts of HT-29 cells significantly accumulated after treatment with LER extract for 24 h as a dose-dependent manner. The level of Bcl-XL mRNA was decreased gradually after exposure to 50, 100, and 200 µg/mL of LER extract. Furthermore, Bax expression was up-regulated, whereas that of Bcl-2 was down-regulated after exposure to increasing concentrations of LER extract. As the Bax and Bcl-2 expression ratio has been recognized as a key factor in regulation of the apoptotic process [21], we examined the Bax/Bcl ratio after treatment with various concentrations of LER extract. The results showed the Bax/Bcl-2 ratio was sharply increased from 1.6 to 54.1 (Figure 4D). These experiments revealed that the increased Bax/Bcl-2 ratio accelerated apoptosis induced by LER extract in HT-29 cells.

### 2.5. Discussion

Anti-tumor activity is estimated as the capacity of the test sample to selectively inhibit the growth of certain cancer cell lines [8]. Therefore, an agent that can selectively induce cell death in cancer cells with low cytotoxicity to the normal cells would be a potential chemotherapeutic agent against cancer. In this study, the antiproliferative effect of LER extract was assessed by the MTT assay. Mitochondrial dehydrogenase in living cells reduces yellow MTT to blue-purple formazan crystals [22]. We tested the anti-cancer effect of LER extract on human colon cancer cells (HT-29) and human hepatocellular liver carcinoma cells (HepG2). The anti-cancer effect of LER extract on HT-29 cells was much better compared to HepG2 cell lines (Figure 1B). Thus we focused on the anti-cancer effect on HT-29 cells in our further study. As shown in Figure 1C, all concentrations of extract we tested showed good inhibition effects on the proliferation of HT-29 cells in a time-dependent manner. Before the treatment for 24 h, the cell viability was sharply decreased. The 48 and 72 h incubated groups exhibited no obvious changes compared with the 24 h incubation group. Furthermore, the cytotoxicity of LER extract to a normal cell line (CCD-986Sk) was examined, and no obvious toxicity was observed when the cells were treated for 200 µg/mL LER extract for 24 h (Figure 1A). These findings provided the impetus for further studies on the mechanism(s) action of the LER extracts.

Apoptosis is the result of a highly complex cascade of cellular biochemical events characterized by blebbing, cell shrinkage, nuclear fragmentation, and chromatin condensation [23]. Most anti-cancer agents exert their cytotoxic effects by inducing apoptosis in tumor cells [24]. Therefore, the activity of apoptosis induction is vital in cancer treatment, and several chemotherapeutic drugs have been shown to induce apoptosis *in vitro* [25]. In this study, the treatment of LER extracts severely distorted the cellular morphology of HT-29 cells (Figure 1D). When the dose up to 200 µg/mL, the majority of cells exhibited morphological features typical of apoptotic cells, including obvious shrinkage, destruction of the monolayer, and condensed chromatin. These experiments suggest that LER has anticancer properties in colon cancer cells via induction of apoptosis.

Cell growth is controlled by several genetically defined checkpoints which ensure the progression of cells through different stages of the cell cycle [26]. Some anticancer drugs inhibit cell proliferation and induce apoptosis via activating a variety of intracellular signaling pathways that arrest the cell cycle in the G1, S, or G2 phases [27]. It was also reported that the G2/M checkpoint plays an important role for DNA damage-induced apoptosis. It has been reported that most anticancer drugs induced cancerous DNA damage, blocked mitosis and arrested cells at the G2/M phase [28,29,30]. The G2/M phase cell cycle arrest involves interactions targeting tubulin or disrupting the tubulin-microtubule equilibrium [31]. In this study, the cells at G2/M phase after treatment by LER was almost 4 times higher compared to untreated control cells, which indicates that G2/M arrest by LER might participate in the inhibition of microtubule dynamics. Additional studies are needed to investigate effectively the molecular mechanism arresting HT-29 cells in the G2/M phase of the cell cycle.

In living organisms, a moderate increase in ROS can promote cell proliferation and differentiation, however, excessive amounts of ROS can cause oxidative damage to cells, and also be toxic to cancer cells [32]. Mitochondria are a source of ROS and play a central role in the regulation of apoptotic signaling. ROS, as a second messenger in multiple signaling pathways, play an important role in apoptosis by regulating the activity of certain enzymes involved in the cell death pathway [33]. Some anticancer drugs have been reported to trigger apoptosis in tumor cells, in part by inducing the formation of ROS [34]. In present study, the ROS level in HT-29 cells treated by LER extract was increased compared to control cells, which indicated that the LER-induced apoptosis in HT-29 cancer cells require the generation of ROS and LER extract induced apoptotic cell death through a ROS-dependent mechanism. Increased ROS product was suggested to be associated with mitochondria injury with a marked decreased in MMP [35]. Thus, we examined whether LER-induced ROS production in HT-29 cells could be coincident with changes in MMP during apoptosis (Figure 3). LER treatment led to a rapid drop in MMP measured as a decrease in fluorescence. A high level of ROS in cells can cause peroxidative damage to the cell membrane [36], consequent changes to the membrane permeability [37], and loss of MMP. The major functions of mitochondria are protein import, adenosine triphosphate (ATP) generation, and oxidative phosphorylation redox reactions [38]. The MMP loss during apoptosis may be contribute to the death cell through the loss of mitochondrial functions.

Cells undergo apoptosis through two major pathways, including the extrinsic death receptor pathway, which triggers the activation of a caspase cascade, and the intrinsic mitochondrial pathway [39]. Caspases are closely involved in extrinsic apoptotic cell death and caspase-3 is the most commonly activated caspase during apoptosis [40]. In this study, the expression of caspase-3 was sharply induced by LER treatment in a dose-dependent manner, which confirms the conclusion that the LER can induce apoptosis in HT-29 cells (Figure 4A). Some scientists have reported that the apoptosis process involves upregulation of *caspase-3* mRNA, cleavage and activation of the inactive caspase-3 and loss of the activated caspase 3. Charles *et al*. [41] found the activation of caspase-3 during apoptosis could be a direct consequence of enhanced oxidative stress (e.g., generation of ROS). The reduction of oxidative stress by treatment with SOD suppresses activated transcription of caspase-3, thus leading to decreased levels of transcription of the mRNA. In our study, ROS level was gradually increased accompanied with the increase of caspase-3 transcripts level (Figure 3 and Figure 4), which supported the conclusion of Charles *et al*. [41]. So we thought the increase of caspase-3 mRNA was caused by increase ROS generation during the process of apoptosis in HT-29 cells treated by LER extract of different concentrations. Bcl-2 family proteins contain two types, including pro-apoptotic proteins such as Bax, Bak, and Bcl-Xs and the other type is antiapoptotic proteins such as Bcl-2, Bcl-XL [42]. Both biochemical and genetic evidence indicates that Bcl-2 family members can regulate cell death induced by caspases [43]. Bcl-2, a caspase substrate, can function as an antioxidant to inhibit apoptosis in a wide variety of cell types, which play a vital role in regulating the mitochondria-dependent pathway [44]. Previous studies have demonstrated that Bcl-2 and Bax locate in the mitochondrial outer-membrane and the Bcl-2/Bax ratio can be recognized as a key factor in regulation of the apoptotic process due to it regulates the release of mitochondrial cytochrome *c* to cytosol [21]. Our results showed that LER promoted the expression of Bax (pro-apoptotic protein) and decreased the levels of Bcl-2 and Bcl-XL (anti-apoptotic proteins) in a dose-dependent manner. Furthermore, the Bax/Bcl-2 ratio was sharply increased from 1.6 to 54.1 (Figure 4B). Therefore, these results indicate that treatment with LER induced cell death in mitochondrial apoptosis pathway.

## 3. Experimental

### 3.1. Plant Material and Extract Preparation

LER fruits was air dried under shade at room temperature and then powered. Ten grams of sample were extracted with methanol (200 mL) for 72 h. The extracts were filtered and evaporated in a rotary evaporator at 50 °C to obtain the final extract. The extract was dissolved in dimethyl sulfoxide (DMSO) and the final concentration of DMSO in each sample is 0.1%. The “zero” group in each experiments is the control group which was treated with 0.1% DMSO.

### 3.2. Cell Lines and Cell Culture

Cortical collecting duct (CCD-986Sk) cell lines were grown in Dulbecco’s Modified Eagle’s medium (DMEM). Human hepatocellular liver carcinoma cells (HepG2) and human colon cancer cell (HT-29) lines were maintained in Roswell Park Memorial Institute medium 1640 (RPMI 1640). The media were supplemented with 10% (v/v) heat-inactivated fetal bovine serum, 100 U/mL penicillin and 100 µg/mL streptomycin. Cells were cultured in a humidified atmosphere and incubated at 37 °C in 5% CO_2_.

### 3.3. Cytotoxicity and Anti-Proliferation Test

CCD-986Sk cell line was used as normal cells control in this study. This cell line belongs to fibroblast cells and was established from skin taken from normal breast tissue removed during breast reduction mammoplasty. CCD-986Sk cell lines were seeded in 96-well plates (1 × 10^5^ cells/well) and incubated in DMEM, at 37 °C in a 5% CO2 for 24 h. The cells were pretreated with 100, 200, 400, and 800 µg/mL of LER for 24 h. Otherwise, HepG2 and HT-29 cells were seeded in 96-well plates (1 × 10^5^ cells/well) and incubated in RPMI 1640, at 37 °C in a humidified atmosphere of 5% CO_2_ for 24 h. The two cell lines were incubated with 50, 100, and 200 µg/mL of LER extracts respectively for 24 h. In addition, The HT-29 cells were incubated with 50, 100, and 200 µg/mL of LER extracts for 6, 12, 24, 48, 72 h, respectively. A 2 mg/mL MTT solution (20 µL) was added to each well and incubated. After 4 h of incubation, the supernatant was discarded and 200 μL of DMSO was added to each well to terminate the reaction. The absorbance was measured at 550 nm using an enzyme-linked immunosorbent assay (ELISA) plate reader (Bio-Tek, Winooski, VT, USA). For the treated cells, viability is expressed as the percentage of control cells.

### 3.4. Light Microscopic Examination

HT-29 cells were seeded in 6-well plates (1 × 10^5^ cells/well) and incubated for 24 h. After treated with 50, 100, and 200 µg/mL of LER extracts for further 24 h, the cells were observed under a light microscope (Olympus, Japan).

### 3.5. Cell Cycle Analysis

The proportion of HT-29 cancer cells in different phases were measured by flow cytometry analysis. HT-29 cells were seeded in 6-well plates (1 × 10^6^ cells/well) for 24 h. Then, the cells were incubated with 50, 100, 200 μg/mL of LER extracts for 24 h. Collected the cells and washed twice with phosphate buffered saline (PBS). Each group (1 × 10^6^) was fixed in 1 mL fixative at −20 °C for 2 h. Following decanted the fixative, the cell pellet was suspend in 0.5 mL staining solution with propidium iodide (PI). After incubated for 30 min at room temperature in the dark, the cells were analyzed by flow cytometry (Becton-Dickinson, Franklin Lakes, NJ, USA).

### 3.6. Measurement of Reactive Oxygen Species (ROS) Level

The level of intracellular ROS was measured by the change in fluorescence resulting from oxidation of dichlorofluorescin diacetate (DCFH-DA). Cells were seeded in 6-well plates (1 × 10^6^ cells/well) for 24 h. After incubation, the cells were treated with different concentration of LER extracts for 24 h. Then, the cells were detached by trypsin-EDTA and washed with PBS. Cells were treated with 20 µM dichlorofluorescin diacetate (DCFH-DA) for 30 min in the dark. Intracellular ROS were measured via a FACS Calibur flow cytometer (Becton Dickinson, USA), with an excitation filter of 485 nm and an emission filter 535 nm. Fluorescence was measured via the FACS Calibur flow cytometer, with an excitation filter of 485 nm and an emission filter 535 nm, and data were analyzed with the Cell Quest program.

### 3.7. Measurement of Mitochondrial Membrane Potential (MMP) Level

The mitochondrial membrane potential level was measured by 3,3'-dihexyloxacarbocyanine iodide (DiOC_6_). Briefly, HT-29 cells (1 × 10^6^) were seeded in 6-well plate for 24 h. Followed treatment of different concentration of LER, cells were washed with PBS Cells and incubated with DiOC_6_ (20 nm) at 37 °C for 20 min. Collected the cells and washed again with PBS and resuspended in PBS. Loss of mitochondrial membrane potential was detected in cells by measuring a change in fluorescence by FACS Calibur flow cytometer, with excitation at 484 nm and emission at 501 nm.

### 3.8. RNA Isolation and Reverse Transcription-Polymerase Chain Reaction (RT-PCR) Analysis

Total RNA was isolated from the LER treated cells by using a Trizol RNA isolation kit (Invitrogen, Carlsbad, CA, USA) and stored at −80 °C for subsequent analysis. First-strand cDNA was synthesized from 1 μg of the DNase-treated total RNA using AccuPower^®^ PCR PreMix containing oligo(dT) primers and Moloney murine leukemia virus reverse transcriptase (M-MLV RT, Invitrogen). The sequences of primers used in RT-PCR were shown in Table 1. The conditions for caspase-3, Bcl-XL, and Bcl-2 were 94 °C for 5 min and the amplification was followed by 30 cycles of denaturing at 94 °C for 30 s, annealing at 60 °C for 30 s, and primer extension a 72 °C for 45 s with a final extension at 72 °C for 7 min. The PCR conditions for Bax and β-actin were 94 °C for 5 min and the amplification was followed by 25 cycles of denaturing at 94 °C for 30 s, annealing at 55 °C for 30 s, and primer extension a 72 °C for 45 s with a final extension at 72 °C for 7 min. The PCR product was analyzed on 1% agarose gels and DNA bands were visualized by ethidium bromide staining and a Mini BIS image analysis system (DNR Bio-Imaging Systems Ltd., Kiryat Anavim, Israel). All experiments were performed in triplicate.

**Table 1:** The sequences of primers used in RT-PCR.

Primers	Forward primer (5'–3')	Forward primer (5'–3')
capspase-3	TCACAGCAAAAGGAGCAGTTT	CGTCAAAGGAAAAGGACTCAA
Bcl-XL	CCAGAAGGGACTGAATCG	CCTTGTCACGCTTTCCAC
Bax	TCCACCAAGAAGCTGAGCGA	GTCCAGCCCATGATGGTTCT
Bcl-2	TGTGGCCTTCTTTGAGTTCG	TCACTTGTGGCTCAGATAGG
β-actin	TCACCCTGAAGTACCCCATC	CCATCTCTTGCTGCAAGTCC

### 3.9. Statistical Analyses

Cytotoxicity and anti-proliferative activity of LER extract were carried out independently in triplicate (*n* = 3). Data are expressed as the mean ± standard derivation (SD). One-way analysis of variance (ANOVA) was used to test for significant differences between the groups followed by the Duncan test for multiple comparisons. Differences were considered significant when *p* < 0.05. All analyses were performed using SPSS 16 (SPSS Institute, Cary, NC, USA). Gene expression levels were quantified with the image analysis software program (Quantity One; Bio-Rad, Hercules, CA , USA).

## 4. Conclusions

In conclusion, our study indicates that the methanolic extract of LER exerts its antiproliferative effects by inducing apoptotic cell death, and causing G2/S arrest in HT-29 cells. LER also promotes ROS generation and MMP loss in mitochondria and regulates the Bax and Bcl-2 genes. Thus, *L. echinata* Roxb. should be considered as a potential therapeutic in the treatment of human colon cancer. However, additional studies to determine its *in vivo* biological activities and to identify the phytochemicals responsible for their anticancer activities will be needed.

## References

[1] Feeley T.H., Cooper J., Foels T., Mahoney M.C. (2009). Efficacy expectations for colorectal cancer screening in primary care: Identifying barriers and facilitators for patients and clinicians. Health Commun..

[2] Urruticoechea A., Alemany R., Balart J., Villanueva A., Vinals F., Capella G. (2010). Recent advances in cancer therapy: An overview. Curr. Pharm. Des..

[3] Matsuhashi N., Saio M., Matsuo A., Sugiyama Y., Saji S. (2005). Apoptosis induced by 5-fluorouracil, cisplatin and paclitaxel are associated with p53 gene status in gastric cancer cell lines. Int. J. Oncol..

[4] Hirao M., Fujitani K., Tsujinaka T. (2004). Phase I study of the combination of nedaplatin, adriamycin and 5-fluorouracil for treatment of advanced esophageal cancer. Dis. Esophagus.

[5] Legarza K., Yang L.X. (2006). New molecular mechanisms of action of camptothecin-type drugs. Anticancer Res..

[6] Slater T.F., Sawyer B., Strauli U.D. (1963). Studies on succinate-tetrazolium reductase systems. III. Points of coupling of four different tetrazolium salts. Biochim. Biophys. Acta.

[7] Fulda S., Debatin K.M. (2003). Apoptosis pathways in neuroblastoma therapy. Cancer Lett..

[8] Hu W., Lee S.K., Jung M.J., Heo S.I., Hur J.H., Wang M.H. (2010). Induction of cell cycle arrest and apoptosis by the ethyl acetate fraction of *Kalopanax pictus* leaves in human colon cancer cells. Bioresour. Technol..

[9] Kirtikar K.R., Basu B.D. (1933). Indian Medicinal Plants.

[10] Chaudhary G.R., Sharma V.N., Siddiqui S.  (1951). Amariin: A bitter constituent of *Luffa* species.

[11] Bhatt R.H., Khurana M.L. (1957). Studies on *Luffa echinata*. Indian J. Pharm..

[12] Khorana M.L., Raisinghani K.H. (1961). Studies of *Luffa echinata* III. The oil and the saponin. J. Pharmacol. Sci..

[13] Lavie D., Shvo Y., Gottlieb O.R., Desai R.B., Khorana M.L. (1962). The occurrence of 2-epicucurbitacin B in *Luffa echinata*. J. Chem. Soc..

[14] Mesbah U.A., Haque M.E., Sutradhar R.K. (1994). Bitter principe of *Luffa echinata*. Phytochemistry.

[15] Lauria P., Sharma V.N., Vanjani S., Sangal B.C. (1976). Role of *Luffa echinata* in liver injury and its other pharmacological actions. Indian J. Pharmacol..

[16] Bhatt R.H., Khorana M.L., Gaitonde B.B., Raiker K.P., Patel J.R., Kekre M.S. (1958). Diuretic properties of saponin of *Luffa echinata* Roxb. J. Gr. Hosp. Grant. Med. Coll..

[17] Aswal B.S., Bhakuni D.S., Goel A.K., Kar K., Mehrotra B.N., Mukherjee K.C. (1984). Screening of Indian plants for biological activity. Part X. Indian J. Exp. Biol..

[18] Nadkarni K.M. Dr. K. M. (1976). Nadkarni’s Indian Materia Medica, Part III.

[19] Bhatt R.H., Khorana M.L., Patel J.R., Gaitonde B.B., Kekre M.S. (1958). Pharmacological studies of saponins of the fruits of *Luffa echinata* Roxb and seeds of *Trigonella foenum-graecum* Linn. Indian J. Physiol. Pharmacol..

[20] Ahmed B., Alam T., Khan S. (2001). Hepatoprotective activity of *Luffa echinata* fruits. J. Ethnopharmacol..

[21] Yang H.L., Chen C.S., Chang W.H., Lu F.J., Lai Y.C., Chen C.C., Hseu T.H., Kuo C.T., Hseu Y.C. (2006). Growth inhibition and induction of apoptosis in MCF-7 breast cancer cells by *Antrodia camphorata*. Cancer Lett..

[22] Mossman T. (1983). Rapid colorimetric assay for cellular growth and survival: Application to proliferation and cytotoxicity assays. J. Immunol. Methods.

[23] Debatin K.M., Krammer P.H. (2004). Death receptors in chemotherapy and cancer. Oncogene.

[24] Kaufman S.H. (1989). Induction of endonucleolytic DNA cleavage in human acute myelogenous leukemia cells by etoposide, camptothecin and other cytotoxic anticancer drugs: A cautionary note. Cancer Res..

[25] Huschtscha L.I., Bartier W.A., Ross C.E.A., Tattersall M.H. (1996). Characteristics in cancer death after exposure to cytotoxic drugs *in vitro*. Br. J. Cancer.

[26] Hoyt M.A. (2004). A new checkpoints take shape. Nat. Cell Biol..

[27] Kumar N., Afeyan R., Kim H.D., Lauffenburger D.A. (2008). Multipathway model enables prediction of kinase inhibitor cross-talk effects on migration of Her2-overexpressing mammary epithelial cells. Mol. Pharmacol..

[28] Hsu S.C., Ou C.C., Li J.W., Chuang T.C., Kuo H.P., Liu J.Y., Chen C.S., Lin S.C., Su C.H., Kao M.C. (2008). Ganoderma tsugae extracts inhibit colorectal cancer cell growth via G2/M cell cycle arrest. J. Ethnopharmacol..

[29] Wang J., Wu A., Xu Y., Liu J., Qian X. (2009). M2-A induces apoptosis and G2-M arrest via inhibiting PI3 K/Akt pathway in HL60 cells. Cancer Lett..

[30] Zhao Y.Y., Shen X., Chao X., Ho C.C., Cheng X.L., Zhang Y., Lin R.C., Du K.J., Luo W.J., Chen J.Y. (2011). Ergosta-4, 6, 8(14), 22-tetraen-3-one induces G2/M cell cycle arrest and apoptosis in human hepatocellular carcinoma HepG2 cells. Biochim. Biophys. Acta.

[31] Hadfield J.A., Ducki S., Hirst N., McGown A.T. (2003). Tubulin and microtubules as targets for anticancer drugs. Prog. Cell Cycle Res..

[32] Boonstra J., Post J.A. (2004). Molecular events associated with reactive oxygen species and cell cycle progression in mammalian cells. Gene.

[33] Wu W.S. (2006). The signaling mechanism of ROS in tumor progression. Cancer Metastasis Rev..

[34] Kamata H., Hirata H. (1999). Redox regulation of cellular signalling. Cell. Signal..

[35] Wang X., Sharma R.K., Gupta A., George V., Thomas A.J., Falcone T., Agarwal A. (2003). Alterations in mitochondria membrane potential and oxidative stress in infertile men: A prospective observational study. Fertil. Steril..

[36] Cummins J.M., Jequiers A.M., Kan R. (1994). Molecular biology of human male infertility: Links with aging, mitochondrial genetics and oxidative stress. Mol. Reprod. Dev..

[37] Kim W.H., Park W.B., Gao B., Jung M.H. (2004). Critical role of reactive oxygen species and mitochondrial membrane potential in Korean mistletoe lectin-induced apoptosis in human hepatocarcinoma cells. Mol. Pharmacol..

[38] Voisine C., Craig E.A., Zufall N., von Ahsen O., Pfanner N., Voos W. (1999). The protein import motor of mitochondria: Unfolding and trapping of preproteins are distinct and separable functions of matrix Hsp70. Cell.

[39] Haupt S., Berger M., Goldberg Z., Haupt Y. (2003). Apoptosis—The p53 network. J. Cell Sci..

[40] Janicke R.U., Sprengart M.L., Wati M.R., Porter A.G. (1998). Caspase-3 is required for DNA fragmentation and morphological changes associated with apoptosis. J. Biol. Chem..

[41] Charles A.K., Hisheh S., Liu D., Rao R.M., Waddell B.J., Dickinson J.E., Rao A.J., Dharmarajan A.M. (2005). The expression of apoptosis related genes in the first trimester human placenta using a short term *in vitro* model. Apoptosis.

[42] Lee W.R., Shen S.C., Lin H.Y., Hou W.C., Yang L.L., Chen Y.C. (2002). Wogonin and fisetin induce apoptosis in human promyeloleukemic cells, accompanied by adecrease of reactive oxygen species, and activation of caspase 3 and Ca^2+^-dependent endonuclease. Biochem. Pharmacol..

[43] Chinnaiyan A.M., O’Rourke K., Lane B.R., Dixit V.M. (1997). Interaction of CED-4 with CED-3 and CED-9: A molecular framework for cell death. Science.

[44] Cheng E.H.Y., Kirsch D.G., Clem R.J., Ravi R., Kastan M.B., Bedi A., Ueno K., Hardwick J.M. (1997). Conversion of Bcl-2 to a Bax-like death effector by caspases. Science.

